# Supplementary material for: Colloidal shuttles for programmable cargo transport
Source: Nat Commun. 2017 Nov 30;8:1872. doi: 10.1038/s41467-017-01956-9 (PMC5709445; doi:10.1038/s41467-017-01956-9)
Supplement: Supplementary file 1 — Supplementary Information [file 41467_2017_1956_MOESM1_ESM.pdf]

Supplementary Information for paper "**Colloidal Shuttles for Programmable Cargo Transport**" by

Ahmet F. Demirörs*, Fritz Eichenseher, Martin J. Loessner and André R. Studart*

E-mail: andre.studart@mat.ethz.ch and ahmet.demiroers@mat.ethz.ch

Supplementary Notes

Analysis of dielectrophoretic forces between particles:

Estimations of the dielectrophoretic forces between shuttle and cargo colloids shown in the main text neglect the influence of the cargo colloids to the electrical field gradients. Here, we conduct a more thorough analysis of the dielectrophoretic forces to show that this simplification is valid when the size ratio between shuttle and cargo is large (Supplementary Figure 8). Cargo particles alter the electric field around themselves and thus develop an electric dipole similar to the one of the colloidal shuttle. To investigate the effect of this electric dipole on the interactions between cargo and shuttle, we performed additional simulations. The results displayed in the Supplementary Figure 8 show the electric field strength around the colloidal shuttle and the cargos when they are apart and after they come closer. The simulations reveal that the electric field alteration around the small cargo colloids changes indeed the field around the larger one but this effect is found to be minimal for the size ratio of the spherical shuttle and cargo particles used in our work. This justifies the simpler calculations assumed in the main text. By contrast, polarization of the cargo may play an important role when more than one circle of cargos is trapped by the shuttle, as is the case in the size-selective experiments shown in Figure 3e,f (main text). In this more complex configuration, the DEP forces around the colloidal shuttle are significantly altered after the first circle of cargos are attracted and trapped by the shuttle. This lowers the attraction for a possible second circle of cargos, which we experimentally observed at high external fields (Figure 3e, f, main text).

Besides the simulations shown in the Supplementary Figure 8, a qualitative analysis of the dipolar interactions between cargo and shuttle colloids is also possible using a simple analytical model that describes the interaction potential between dipole moments. Such dipolar interactions are angle dependent. Attraction between the dipole of the colloidal shuttle and that of the cargo is only possible at the top and the bottom of the colloidal shuttle, which agrees with our description based on DEP forces. When two particles are electrically polarized and exhibit a dipole they interact through dipolar interactions given by

$$u_{dip}(r_{ij}) = 4kT\gamma\left(\frac{a}{r_{ij}}\right)^3\left(1 - 3cos^2\theta_{ij}\right),$$

where a is the radius of the particles, $\mathbf{r}_{ij}$ is the vector between particles i and j, and θ_{ij} is the angle between the vector $\mathbf{r}_{ij}$ and the electric field direction, k is the Boltzmann constant and T is the temperature. $\gamma = \frac{\boldsymbol{p}^2}{16\pi\varepsilon_m a^3 kT}$, where $\boldsymbol{p} = 4\pi\alpha\varepsilon_m a^3\mathbf{E}_{loc}$ is the dipole moment induced by the local electric field $\mathbf{E}_{loc}$= $\mathbf{E}$+$\mathbf{E}_{dip}$. Here, $\mathbf{E}$ is the external electric field and $\mathbf{E}_{dip}$ is the field induced by other dipoles. The term $\left(1 - 3cos^2\theta_{ij}\right)$ in the equation above quantifies the angle dependence of the interaction between two dipoles. When two dipoles are arranged such that $\theta_{ij} > \sim 54.74$ then these dipoles repel each other. If the angle θ_{ij} is below 54.74 then the two dipoles attract each other, making a head-to-toe configuration more favorable. Supplementary Figure 8b displays simplistic sketches of these attractive and repulsive interactions.

Supplementary Figures

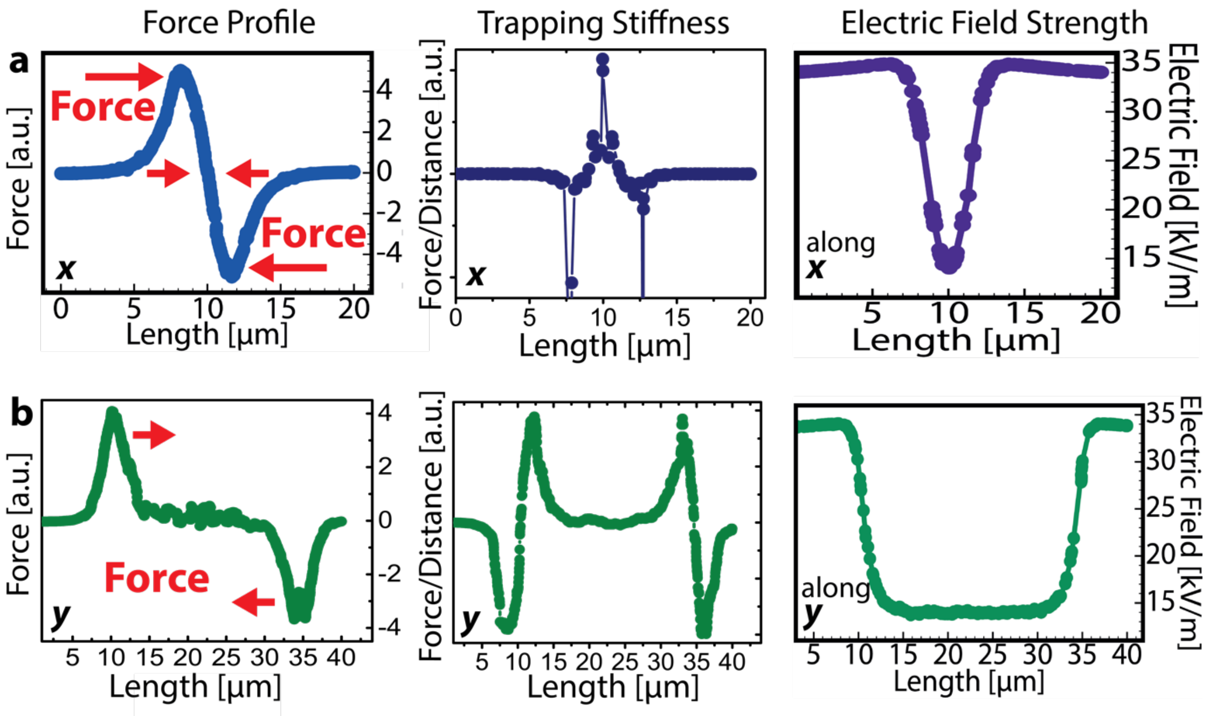

Supplementary Figure 1 | FEA simulations for a silica particle cargo trapped along the (a) short and (b) long axis of a rod shaped colloidal shuttle. (**a**) Along the short axis of the rod (*x*), the size of the cargo particle is comparable to the diameter of the rod. Thus, the cargo will be tightly confined within the center of the trap where the trapping stiffness peaks. This position corresponds to **F**=0 in the force profile and to the minimum in the electric field strength. (**b)** Along the long axis of the rod (*y*), restoring forces will only be present at the edges of the rod. Because of the nearly neutral forces acting in the middle of the rod, the cargo particle will be free to move and exhibit Brownian motion along the long axis of the rod. In this case, the trapping stiffness is maximum at the edges, which means that particles with a comparable size to the short axis will spend more time at the edges compared to the center of the rod.

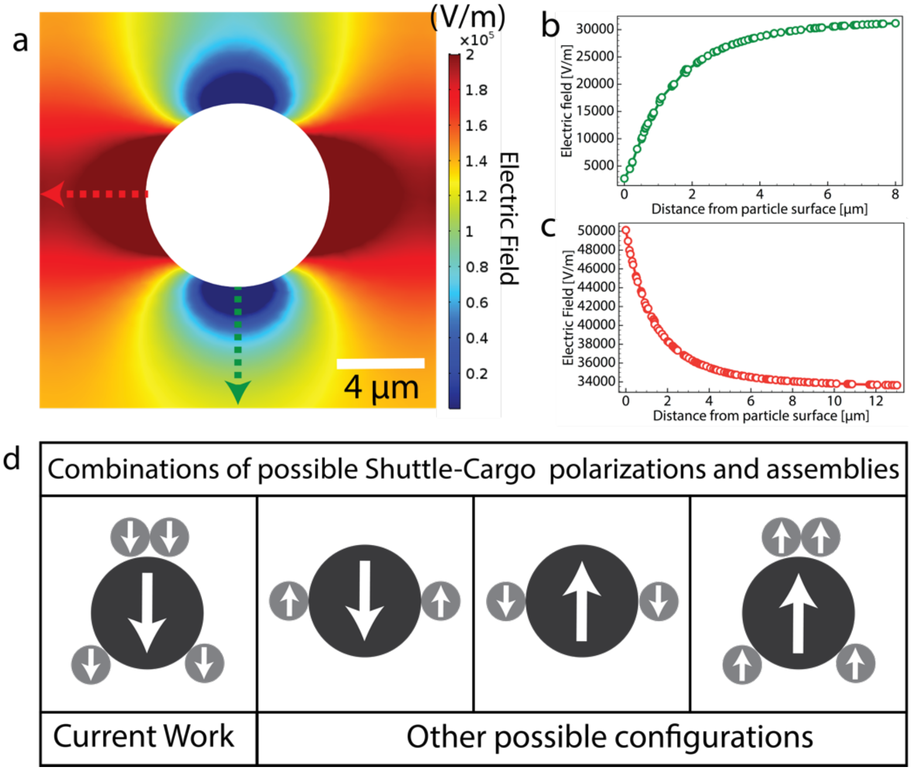

Supplementary Figure 2 | Electric polarization induced by a colloidal shuttle under a uniform electric field and combinations of shuttle-cargo assemblies for different polarizations. (**a**) The colloidal shuttle polarizes the surroundings to form an electric dipole. A negative dielectric contrast ($\varepsilon p < \varepsilon m$) is assumed in this calculation. This leads to a negative polarization, which is shown as an arrow pointing down in (d). Low field regions are generated on the top and bottom while high field regions arise on the sides. This polarization effect will not differ much if the particle is situated close to the electrode surface (Fig. 1, main text). (**b, c**) The gradient in electric field around the colloidal shuttle spans over a length scale that is on the order of the colloid diameter, which was 8 μm in the calculations. Field strength values are shown in V m^{-1}. (**d**) Combinations of possible shuttle and cargo polarizations and types of assemblies depending on their polarization. This shows that provided they are polarized the cargo and shuttle will always interact but the position of the assembly will vary.

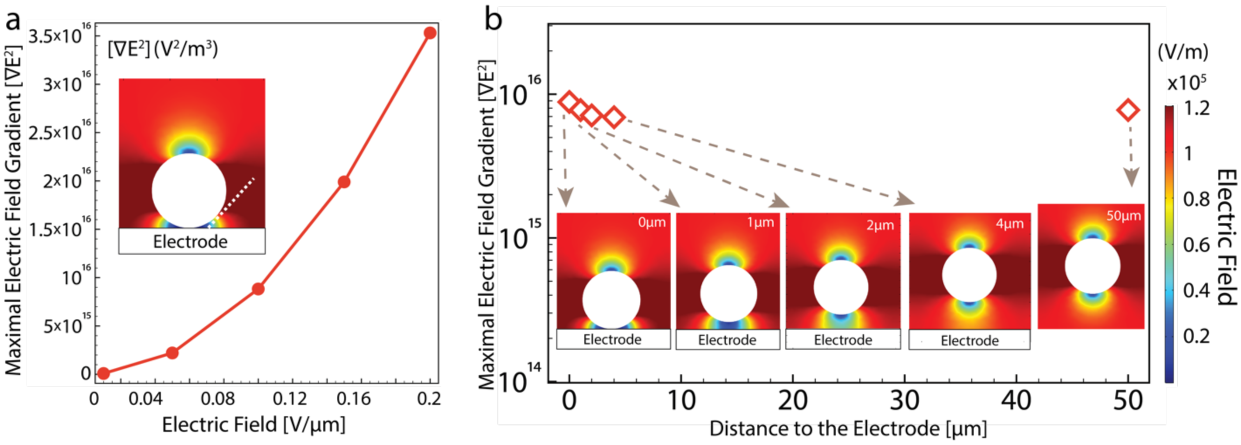

Supplementary Figure 3 | Variation of the electric polarization of a colloidal shuttle with the elevation of the shuttle from the electrode surface. (**a**) From the finite element analysis, we estimated the maximal field gradient around the shuttle by measuring the change in electric field strength along the line shown in the inset. Here, we plotted the maximal gradient of the electric field square ($\nabla \mathbf{E}^2$) value around the colloidal shuttle as a function of electric field applied between the electrodes. This $\nabla \mathbf{E}^2$ value is a direct measure of the $\mathbf{F_{DEP}}$ ($\mathbf{F}_{\mathrm{DEP}} = 2\pi a^3 \varepsilon_m \mathrm{Re}\left\{\frac{\varepsilon_m^* - \varepsilon_p^*}{\varepsilon_m^* + 2\varepsilon_p^*}\right\} \nabla|\mathbf{E}|^2$). (**b**) Analyses of the field gradients around the colloidal shuttle were performed as a function of distance from the electrode until 50 μm, which corresponds to the middle between the two electrode surfaces. Except for a stronger polarization in the bottom side of the shuttle at distances very close to the electrode surface, the estimated maximal $\nabla \mathbf{E}^2$ stays essentially constant along different elevations. This means that the $\mathbf{F_{DEP}}$ forces will stay constant within the sample cell, proving that our technique operates in 3D with the same efficiency as on the surface of the electrode. A negative dielectric contrast ($\varepsilon p < \varepsilon m$, negative polarization) is assumed in these calculations.

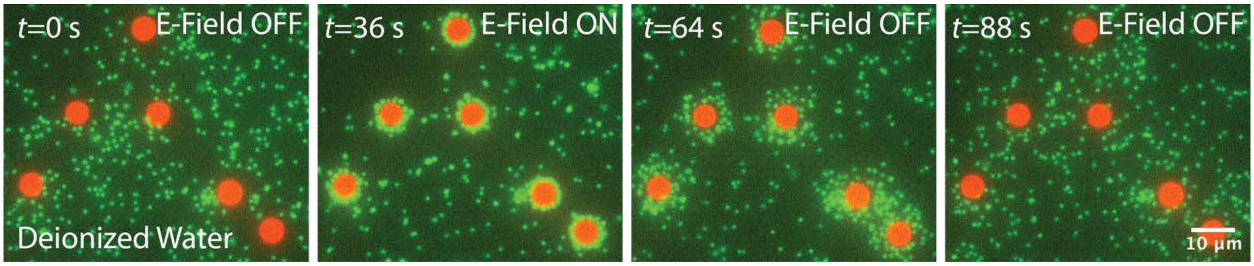

Supplementary Figure 4 | Confocal microscopy images of magneto-dielectric (red) colloids and the reversible assembly of 1 μm sized colloids around the colloidal shuttle with the application of electric field (E=0.05 V μm^{-1}). Here shuttle and cargo colloids were suspended in deionized water.

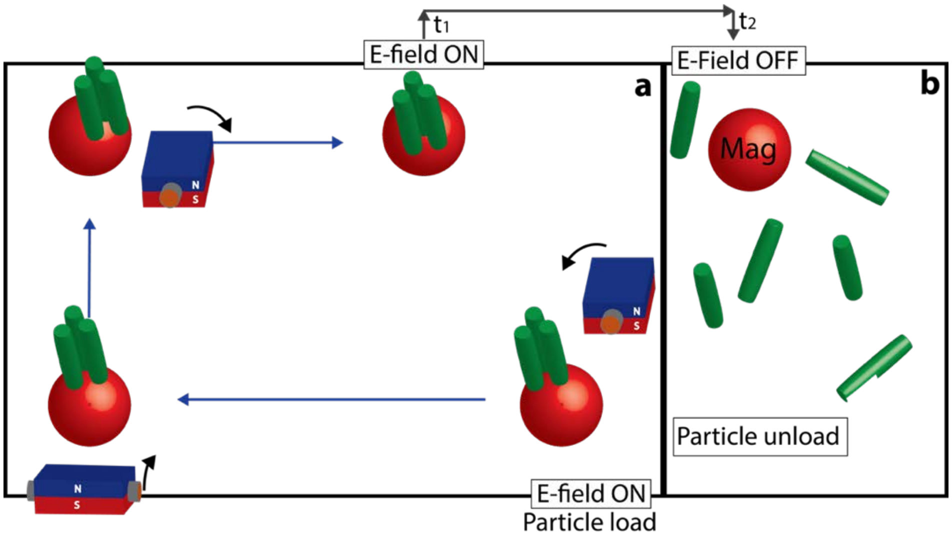

Supplementary Figure 5 | Magnetic rotation set up used to pick up, transfer and release E-coli. Control of the direction of motion for the shuttle-cargo assembly was established by changing the plane of rotation for the permanent magnet, which was attached to a rotating motor. Cartoon (a) shows the pick-up and transfer events, while (b) depicts cargo release.

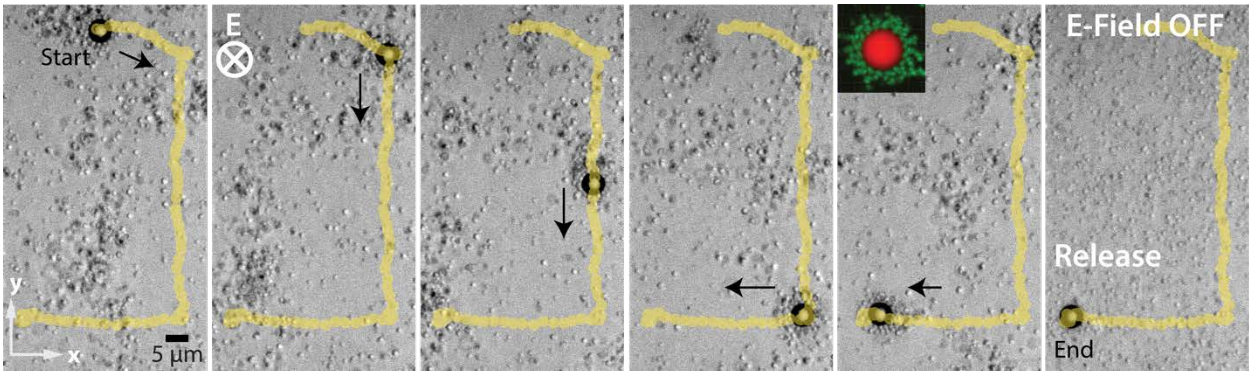

Supplementary Figure 6 | Precise control of the trajectory the colloidal shuttle and cargo. To demonstrate such level of control we used model silica particles as cargo and used magnetic rolling to transfer the cargo colloids. The direction of the shuttle was purely depended on the rotation-direction of the magnet, which we used to drive our clusters in space as shown above in Supplementary Fig. 4**.** Control of the direction of motion for the shuttle-cargo assembly was established by changing the plane of rotation of the magnet.

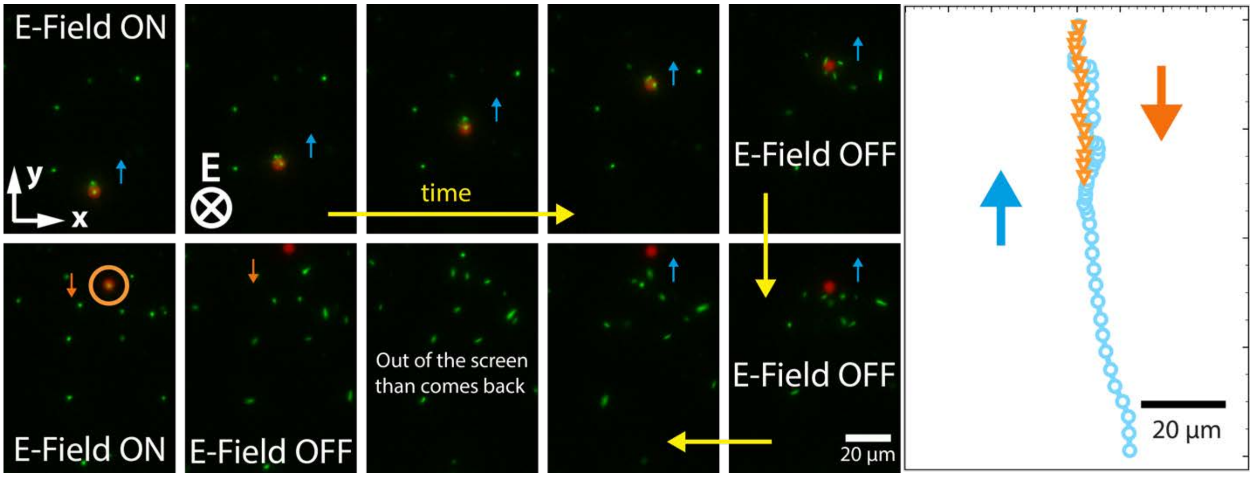

Supplementary Figure 7 | Reversible loading, transport and unloading of E-coli bacteria by a superparamagnetic colloidal shuttle. The motion of the colloidal shuttle and the E-coli cargo is shown in snapshots. In the upper row, the colloidal shuttle is moving upwards. The lower row depicts the release of the bacteria followed by its re-capture when the trajectory of the shuttle is reversed and the electrical field is turned on again (from right to left). The plot on the right displays the trajectory of the shuttle upwards (blue) and downwards (orange).

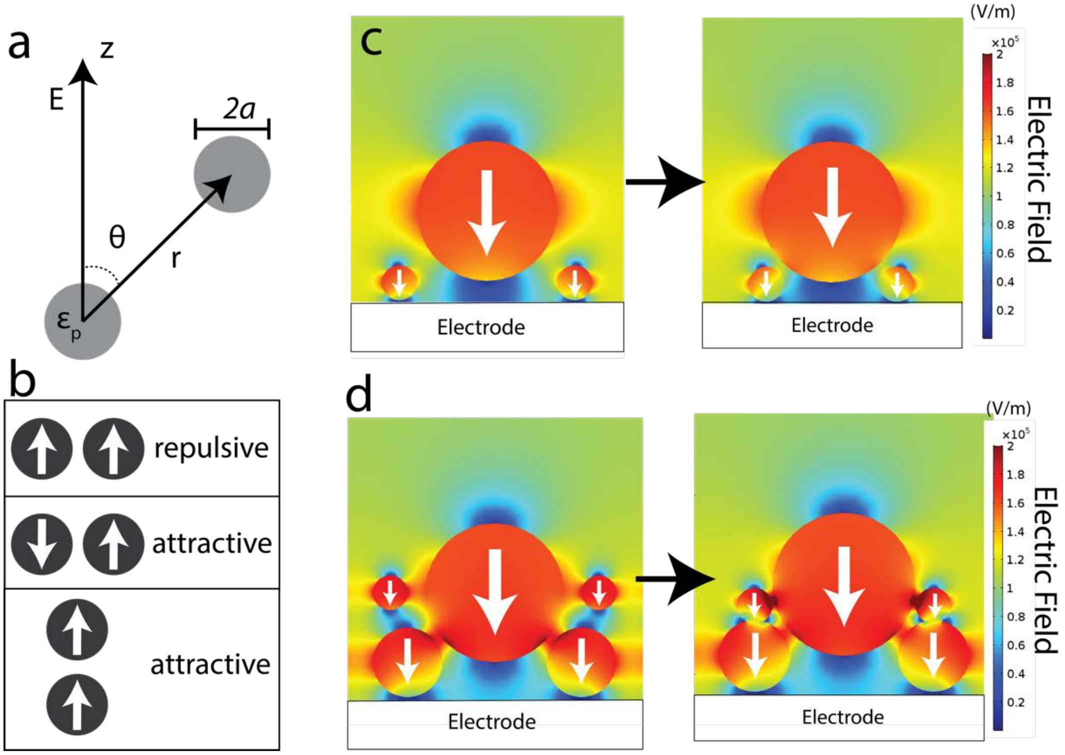

Supplementary Figure 8 | Finite Element Analyses of the electric field around the shuttle before and after the formation of the shuttle-cargo assembly. A shuttle-cargo size ratio similar to the experiments was used in these simulations. To aid visualization of the interactions between many polarized particles, we indicate with an arrow the dipoles induced on the colloids due to the applied electric field. Assuming a negative dielectric contrast ($\varepsilon_p < \varepsilon_m$) for the shuttle and cargo colloids, the particles will become negatively polarized, as indicated by an arrow pointing

down. (**a**) Sketch of colloids interacting via dipole-dipole interactions. (**b**) Summary of how two dipolar particles will behave depending on the angle θ between them. (**c**) First circle of cargos (1 μm) before and after (right) approaching the shuttle, indicating minimum distortion of the electrical dipoles when the cargo is in close proximity to the shuttle particle. (**d**) Second circle of cargos (1μm) before and after approaching a shuttle that had already been surrounded by larger (2 μm) cargo particles. This situation represents the configuration observed in the experiment described in Figure 3e,f (main text). The simulations reveal strong distortions of the electric field around particles if multiple particles are close to one another. Note here that in the assembly of a second circle of cargos, the interactions between the multiple particles involved become more complex, which likely affects the pick-up of these new cargos. While the interactions between the cargo of the 2nd and 1st circles are attractive, the shuttle and 2nd circle of cargo may interact repulsively depending on the angle between them. The magnitude of these interactions also change with the sizes of the shuttle and cargos. In addition, such cargo-cargo interactions are also possible when both cargo colloids are 1 μm sized; however, we did not observe them at the typical field strengths (E=0.01- 0.1 V μm^{-1}) used in this work. The dipole moment (***p***) induced by the local electric field ($\mathbf{E}_{loc}$) scales with ~a^3: $\boldsymbol{p} = 4\pi\alpha\varepsilon_m a^3\mathbf{E}_{loc}$, where a is the radius of the particles, $\alpha = \frac{\varepsilon_p-\varepsilon_m}{\varepsilon_p+2\varepsilon_m}$, ε_p is the dielectric constant of the particle and ε_m is the dielectric constant of the medium. Therefore, cargo-cargo assemblies are more likely to occur for larger cargos and/or at higher field strengths.

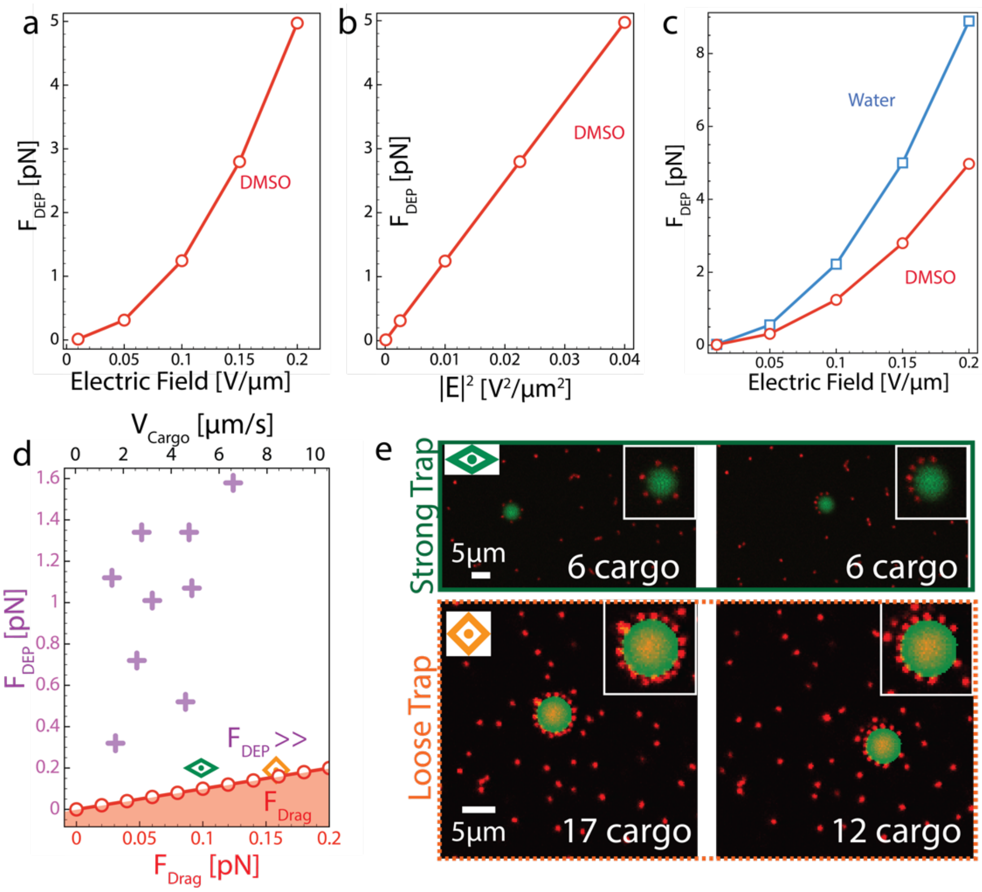

Supplementary Figure 9 | Theoretical and experimental analyses of dielectrophoretic (DEP) trapping forces (F_{DEP}) and the drag forces exert on the colloids due to their motion in liquid medium. DEP forces acting on a 1μm sized silica cargo were estimated by extracting from the finite element analysis the maximal gradient of the electric field square ($\nabla\mathbf{E}^2$) value around the colloidal shuttle as a function of applied electric field between the electrodes. This $\nabla\mathbf{E}^2$ value was used to calculate the DEP forces using the relation: $\mathbf{F}_{\text{DEP}} = 2\pi a^3 \varepsilon_m \mathrm{Re}\left\{\frac{\varepsilon_m^* - \varepsilon_p^*}{\varepsilon_m^* + 2\varepsilon_p^*}\right\} \nabla|\mathbf{E}|^2$. (**a,b**) The theoretical $\mathbf{F_{DEP}}$ is plotted against (a) the applied electric field strength and (**b**) the square of the applied electric field strength. The linear plot allows us to estimate the expected $\mathbf{F_{DEP}}$ for a given electric field applied in our experiments. To compare the $\mathbf{F_{DEP}}$ forces in DMSO and in water we plotted in (**c**) $\mathbf{F_{DEP}}$ estimates for both media. (**d**) From the Stokes drag formula given in the main text we estimated the drag forces exerted on a moving cargo with 1 μm size. Experimental velocities of the colloidal shuttles obtained by image analyses were used in these calculations. Successful and unsuccessful cargo trapping experiments were compared in terms of the $\mathbf{F_{DEP}}$ and $\mathbf{F_{Drag}}$ values expected. For all successful transport cases (✚ and ◈ in (d)) $\mathbf{F_{DEP}}$ exceeds $\mathbf{F_{Drag}}$. However, loose cargo trapping (◈ in (d)) was observed when $\mathbf{F_{DEP}}$ was on the same order as $\mathbf{F_{Drag}}$. These experiments nicely demonstrate that our predictions for $\mathbf{F_{DEP}}$ and $\mathbf{F_{Drag}}$ reflects accurately the experimental observations. (**e**) Confocal microscopy images of the moving shuttles with strong or loose cargo trapping. Note that the cargo was strongly trapped in all experiments plotted with the (✚) sign in (d), for which $\mathbf{F_{DEP}}$ values are much higher than $\mathbf{F_{Drag}}$.
