# Supplementary material for: Colloidal shuttles for programmable cargo transport
Source: Nat Commun. 2017 Nov 30;8:1872. doi: 10.1038/s41467-017-01956-9 (PMC5709445; doi:10.1038/s41467-017-01956-9)
Supplement: Supplementary file 3 — Description of Additional Supplementary Files [file 41467_2017_1956_MOESM3_ESM.pdf]

Description of Additional Supplementary Files

File Name: Supplementary Movie 1
Description: shows a 1 µm spherical silica particle (red) trapped on a silica rod labeled green. The red cargo particle moves along the long axis of the green rod shuttle.

File Name: Supplementary Movie 2
Description: shows standing rod shaped silica particles trapped over silica rods of the same kind lying on the electrode surface. While the electric field is kept on, the standing particles will move up and down along the lying rod.

File Name: Supplementary Movie 3
Description: depicts the pick-up, transfer and release of model silica colloids as a proof of-principle that can be applied to a wide variety of objects. Here, we used a colloidal shuttle that is magneto-dielectric, which allowed for using magnetic field gradients to manipulate the cargo-shuttle assembly.

File Name: Supplementary Movie 4
Description: demonstrates the pick-up, transfer and release of E-coli bacteria as a model bio-colloid cargo. Here, we observed that most bacteria assemble on the top pole (head) of the colloidal shuttle. This is most likely due to the rod shape of the bacteria, which results in alignment under the external field, thus making the top-pole (head) of the colloidal shuttle more suitable for the trapping position.

File Name: Supplementary Movie 5
Description: demonstrates the size-selectivity of the colloidal shuttle, which allows it to carry larger cargo colloids with or without the smaller ones. Here selectivity is achieved by tuning the electric field strength to adjust the trapping DEP forces.

File Name: Supplementary Movie 6
Description: shows the ability to control the trajectory of the colloidal shuttle and its cargo precisely via rolling the superparamagnetic carrier with a rotating permanent magnet. Cargo is relocated in the imaging frame and delivered at the destination by turning the electric field off.

File Name: Supplementary Movie 7
Description: shows the manipulation of shuttle and cargo using bright field and fluorescence imaging, similarly to the experiment depicted in Supplementary Movie 6. The left-handside fluorescence channel exhibits only the cargo colloids, while the right-hand-side bright field channel shows the whole cluster.
